# Ribonuclease J-Mediated mRNA Turnover Modulates Cell Shape, Metabolism and Virulence in *Corynebacterium diphtheriae*

**DOI:** 10.3390/microorganisms9020389

**Published:** 2021-02-14

**Authors:** Truc Thanh Luong, Minh Tan Nguyen, Yi-Wei Chen, Chungyu Chang, Ju Huck Lee, Manuel Wittchen, HyLam Ton-That, Melissa Cruz, Danielle A. Garsin, Asis Das, Andreas Tauch, Hung Ton-That

**Affiliations:** 1 Division of Oral Biology & Medicine, School of Dentistry, University of California, Los Angeles, CA 90095, USA; thanhtruc.luong@gmail.com (T.T.L.); minhtan279@g.ucla.edu (M.T.N.); yiweichen515@g.ucla.edu (Y.-W.C.); jchang@dentistry.ucla.edu (C.C.); 2 Department of Microbiology & Molecular Genetics, University of Texas Health Science Center, Houston, TX 77030, USA; juhuck@kribb.re.kr (J.H.L.); Melissa.R.Fellers@uth.tmc.edu (M.C.); Danielle.A.Garsin@uth.tmc.edu (D.A.G.); 3 NTT Hi-Tech Institute, Nguyen Tat Thanh University, Ho Chi Minh City 70000, Vietnam; 4 Biological Resource Center, Korea Research Institute of Bioscience and Biotechnology, Jeollabuk-do 56212, Korea; 5 Center for Biotechnology (CeBiTec), Bielefeld University, 33615 Bielefeld, Germany; manuelw@cebitec.uni-bielefeld.de (M.W.); tauch@cebitec.uni-bielefeld.de (A.T.); 6 Department of Chemistry, University of California at Irvine, Irvine, CA 92617, USA; tonthath@uci.edu; 7 Neag Comprehensive Cancer Center, Department of Medicine, University of Connecticut Health Center, Farmington, CT 06030, USA; 8 Molecular Biology Institute, University of California, Los Angeles, CA 90095, USA

**Keywords:** *Corynebacterium diphtheriae*, actinobacterium, ribonuclease, RNase J, virulence, *Caenorhabditis elegans*, siderophore, metabolism, tryptophan biosynthesis

## Abstract

Controlled RNA degradation is a crucial process in bacterial cell biology for maintaining proper transcriptome homeostasis and adaptation to changing environments. mRNA turnover in many Gram-positive bacteria involves a specialized ribonuclease called RNase J (RnJ). To date, however, nothing is known about this process in the diphtheria-causative pathogen *Corynebacterium diphtheriae*, nor is known the identity of this ribonuclease in this organism. Here, we report that *C. diphtheriae* DIP1463 encodes a predicted RnJ homolog, comprised of a conserved N-terminal β-lactamase domain, followed by β-CASP and C-terminal domains. A recombinant protein encompassing the β-lactamase domain alone displays 5′-exoribonuclease activity, which is abolished by alanine-substitution of the conserved catalytic residues His^186^ and His^188^. Intriguingly, deletion of DIP1463/*rnj* in *C. diphtheriae* reduces bacterial growth and generates cell shape abnormality with markedly augmented cell width. Comparative RNA-seq analysis revealed that RnJ controls a large regulon encoding many factors predicted to be involved in biosynthesis, regulation, transport, and iron acquisition. One upregulated gene in the ∆*rnj* mutant is *ftsH*, coding for a membrane protease (FtsH) involved in cell division, whose overexpression in the wild-type strain also caused cell-width augmentation. Critically, the ∆*rnj* mutant is severely attenuated in virulence in a *Caenorhabditis elegans* model of infection, while the FtsH-overexpressing and toxin-less strains exhibit full virulence as the wild-type strain. Evidently, RNase J is a key ribonuclease in *C. diphtheriae* that post-transcriptionally influences the expression of numerous factors vital to corynebacterial cell physiology and virulence. Our findings have significant implications for basic biological processes and mechanisms of corynebacterial pathogenesis.

## 1. Introduction

Messenger RNA (mRNA) turnover is crucial in recycling nucleotides and controlling gene expression that permit bacteria to adapt with environmental changes [1,2,3,4]. RNase E, RNase III, and RNase Y are major endonucleases known to be involved in bacterial mRNA processing; these enzymes typically harbor a catalytic domain and an RNA-binding domain [5]. As an essential enzyme in *Escherichia coli* [6], the endonuclease RNase E is part of the multiprotein RNA degradosome complex that also contains PNPase (a 3′-5′ exonuclease), RhlB (DEAD-box family helicase), and enolase [7]. Through its endoribonucleolytic activity, RNase E cleaves polyribosomal mRNAs into small fragments, which are then further degraded into nucleotides by exoribonucleases, such as PNPase, RNase II, and RNase R [8]. The accessory factors RhlB and poly(A) polymerase (PAP) facilitate exoribonucleolytic activity [8]. Importantly, in bacterial species that lack RNase E, including the Gram-positive Firmicutes *Staphylococcus aureus* and *Bacillus subtilis* [9], RNase Y is the functional equivalent of RNase E that was shown to interact with the degradosome partners in vivo [10].

*Bacillus subtilis* possesses two functional homologs of *E. coli* RNase E, named RNases J1 and J2, initially characterized as endoribonucleases [11] and implicated in global mRNA degradation [11,12]. Intriguingly, *Bacillus* RNases J1 and J2 form a complex that exhibits 5′-to-3′ exoribonuclease activity [13]. Another Firmicute having RNases J1 and J2 is *Streptococcus pyogenes* (GAS); unlike *B. subtilis*, both streptococcal RNases are essential for bacterial growth, suggesting non-redundancy of these enzymes in GAS [14]. In *Enterococcus faecalis*, a leading cause of nosocomial infections, only RNase J2 homolog has been reported; deletion of *rnjB*, coding for RNase J2, resulted in reduced transcript levels of pilus genes, which correlates with decreased biofilm formation by the mutant bacteria [15]. Significantly, the *Enterococcus rnjB* mutant is more sensitive to bile salt, as compared to the parental strain, and the mutant is greatly attenuated in organ colonization in an experimental model of infection [16]. In *Streptococcus mutans*, deletion of genes coding for RNases J1 and J2 causes defects in growth, morphology, acid tolerance, natural competence, and biofilm formation [17], and the two RNases were shown to interact with each other [18]. Like the Firmicutes phylum of Gram-positive bacteria, Actinobacteria also possess homologs of RNase J [19]. For instance, *Streptomyces coelicolor* encodes RNase J, a high-resolution crystal structure of which was determined [20]. *S. coelicolor* RNase J forms a homo-tetramer with each protomer comprised of a β-lactamase domain and β-CASP domain, followed by another β-lactamase domain and a C-terminal domain [20]. In *Streptomyces venezuelae*, deletion of *rnj* leads to cell growth and morphology defects [21]. Both *rnj* mutants in *S. coelicolor* and *S. venezuelae* are also defective in antibiotic production [21,22]. These reports suggest that actinobacterial RNase J may be broadly involved in mRNA degradation. Yet, to date whether RNase J is involved in modulating virulence in actinobacterial pathogens, such as *Corynebacterium diphtheriae* and *Mycobacterium tuberculosis*, has not been addressed.

The Gram-positive actinobacterium *C. diphtheriae* is the causative agent of diphtheria, a life-threatening disease caused by a highly potent exotoxin named diphtheria toxin (DT) [23], which is comprised of a catalytic domain A and a host cell transfer-mediating domain B [24]. Though DT is encoded by the *tox* gene within corynebacteriophage β [25], expression of *tox* is regulated by the iron-activated transcriptional repressor DtxR encoded by the bacterial host *C. diphtheriae* [26,27]. Under high-iron conditions, DtxR becomes activated, and it binds to the promoter region of *tox*, repressing *tox* expression; in contrast, when iron is limiting, as within the upper respiratory tract of human host, repression of *tox* by DtxR is relieved, leading to high expression of DT [28]. Importantly, in addition to *tox*, DtxR regulates expression of genes involved in iron and heme transport and siderophore biosynthesis [29,30]. While DT is a major virulence factor of *C. diphtheriae*, pilus adhesins also play a critical role in pathogenesis. The *C. diphtheriae* type strain NCTC 13,129 encodes three types of heterotrimeric pili, classified by their major subunits, i.e., SpaA-, SpaD-, and SpaH-type [31,32,33]. Importantly, in rodent and *Caenorhabditis elegans* models of infection, pilus-less mutants are significantly attenuated [34,35].

We report here functional studies of an uncharacterized protein (DIP1463) in *C. diphtheriae*, predicted to be RNase J (RnJ). By biochemical methods, we demonstrate that the *C. diphtheriae* putative RNase J possesses 5′-3′ exoribonuclease activity confined at the N-terminal β-lactamase domain. Significantly, we show that a mutant devoid of *rnj* is defective in cell growth and morphology in culture and also in bacterial virulence in a nematode model of infection. RNA-seq analysis revealed that *C. diphtheriae* RNase J regulates a large number of transcripts, many of which encode factors involved in bacterial iron acquisition, biosynthesis, and pilus assembly. Thus, RNase J is a major ribonuclease that influences bacterial physiology and virulence in *C. diphtheriae*. Further genetic and biochemical evidence that the diphtheria toxin itself is dispensable for full corynebacterial virulence in the nematode model revealed that the defective virulence of the *rnj* mutant is due to misexpression of some factors other than DT, the nature and mechanisms of which remain to be elucidated.

## 2. Materials and Methods

### 2.1. Bacterial Strains, Plasmids, Media, and Cell Growth

Bacterial strains and plasmids used in this study are listed in Table S2. *C. diphtheriae* was grown in Heart Infusion (HI) Broth (Becton Dickinson, Franklin Lakes, NJ, USA) or on HI agar plates at 30 °C. *E. coli* strains were grown in Luria–Bertani (LB) broth or on LB agar at 37 °C. When necessary, kanamycin (Kan) was added to cultures at 25 μg mL^−1^; ampicillin (Amp) was used at 50 µg mL^−1^ or 100 µg mL^−1^.

To examine cell growth, overnight cultures of *C. diphtheriae* strains were used to inoculate fresh cultures with starting OD_600_ of ~0.1. Bacterial growth was monitored every hour at OD_600_ using Ultrospec 7000 (GE Healthcare, Boston, MA, USA). Results are presented as average of 3 independent experiments performed in duplicate.

### 2.2. Generation of Gene Deletion Mutants in C. diphtheriae

An in-frame, nonpolar deletion mutant of *rnj* was generated according to published protocols [31,36]. Briefly, 1-kb fragments upstream and downstream of *rnj* were PCR-amplified using appropriate primers (Table S3) and cloned into the vector pK19*mobsacB* at EcoRI and BamHI sites. The resulting plasmid was introduced into *E. coli* S17-1, and the cloned fragments were verified by DNA sequencing. *E. coli* S17-1 harboring the deletion plasmid was used for bacterial conjugation with *C. diphtheriae* NCTC13129. Integration of the plasmid into the corynebacterial chromosome was selected with Kan. Selected integrates were grown overnight at 30 °C without antibiotics to induce double-crossover homologous recombination leading to plasmid excision and generating wild-type and mutant alleles. Mutant alleles were selected by colony PCR. The generated *rnj* mutant, together with *E. coli* S17-1 harboring a *dtxR* deletion plasmid [37], was used to create a double mutant lacking both *rnj* and *dtxR* (Table S2).

### 2.3. Recombinant Plasmids

pRnJ—The primer pair pRnJ-SalI/pRnJ-BamHI (Table S3) was used to amplify a DNA fragment containing the *rnj* promoter and *rnj* coding sequence using genomic DNA of *C. diphtheriae* strain NCTC 13,129 as a template. The generated PCR product was gel-purified, digested with SalI-HF and BamHI-HF (NEB, Ipswich, MA, USA), and subsequently ligated into pCGL0243 [38] precut with SalI and BamHI. The resulting plasmid was introduced into *E. coli* DH5α and plasmid DNA was isolated for PCR, restriction enzymes incubation, and sequencing to confirm the cloned sequence. Finally, the plasmid was electroporated into the *C. diphtheriae* ∆*rnj* mutant.

pFtsH—The primer pairs pFtsH-HindIII-A/pFtsH-B and pFtsH-C/pFtsH-BamHI-D (Table S3) were used to PCR-amplify the *spaA* promoter region and the *ftsH* coding sequence, respectively, using genomic DNA of strain NCTC 13129 as a template. Subsequently, overlapping PCR was employed to link the promoter region to the coding sequence, using both PCR products as templates and primers pFtsH-HindIII-A and pFtsH-BamHI-D, according to a published protocol [39]. The generated PCR product was gel-purified, digested with HindIII-HF and BamHI-HF (NEB), and subsequently ligated into pCGL0243. The resulting plasmid was introduced into *E. coli* DH5α, and plasmid DNA was isolated for confirmation by DNA sequencing. Finally, the plasmid was electroporated into the *C. diphtheriae* strain NCTC 13129.

pMCSG-RnJ_981_ and derivatives—An N-terminal region of RnJ encompassing the β-lactamase domain (residues 1-327) was cloned into the expression vector pMCSG7 according to a published ligation-independent cloning method [40]. Briefly, a pair of primers LIC-RnJ_981_-F/LIC-RnJ_981_-R (Table S3) was used to amplify the first 981 nucleotides of *rnj* from genomic DNA of strain NCTC 13129. The amplicon was ligated into pMCSG7 in a step-down annealing reaction, and the resulting plasmid (pMCSG-RnJ_981_) was introduced into *E. coli* DH5α. Successful clones were confirmed by DNA sequencing before introducing into *E. coli* SHuffle cells for protein expression.

To generate alanine-substitution RnJ mutants at His residues 186 and 188, a site-directed mutagenesis method was employed [40] using pairs of phosphorylated primers LIC-H186A-F/LIC-H186A-R and LIC-H188A-F/LIC-H188A-R (Table S3) and pMCSG-RnJ_981_ as a template. Resulting linear PCR products were ligated with T4 ligase (NEB) at 16 °C for 18 h before transforming into *E. coli* DH5α. The mutations were confirmed by DNA sequencing. The generated recombinant plasmids were introduced into *E. coli* SHuffle cells for protein expression.

### 2.4. Protein Purification

Purification of recombinant RnJ proteins was followed according to a published protocol [40]. Briefly, overnight cultures of *E. coli* SHuffle cells harboring pMCSG-RnJ_981_ or its derivatives were used to inoculate fresh cultures, which were grown at 37 °C with shaking until OD_600_ of ~0.8. Protein expression was induced at 30 °C for 6 h with 1 mM of isopropyl β-D-1-thiogalactopyranoside (IPTG; Sigma, St. Louis, MO, USA). Cells were harvested by centrifugation and lysed by French press, and clear lysates were obtained for affinity chromatography using nickel-nitrilotriacetic acid (NTA) beads. Purified proteins were desalted, concentrated, and stored at −80 °C until further experiments.

### 2.5. RT-FeDEx Assay

To determine 5′-exoribonuclease activity, a RT-FexEX assay was performed as previously described [41]. RNA1 and DNA1 (Table S3) synthesized by Integrated DNA Technology (CA, USA) were hybridized, and the duplex (500 nM) was added to reaction buffer (30 mM Tris-Cl pH 8.0, 2 mM MgCl_2_, 50 mM NH_4_Cl, 0.5 mM DTT, 20 µg/mL acetylated bovine serum) containing purified RnJ proteins (100 nM). Exoribonuclease activity was monitored by a T100 Thermal Cycler (Bio-Rad) at absorbance/emission wavelengths of 494/520 nm, respectively, for 15 h. The experiments were repeated twice in triplicates. The presented graph is representative of an experiment with 30 min intervals, prepared by GraphPad Prism 5.0 (La Jolla, CA, USA).

### 2.6. RNA-Seq Analysis

RNA of *C. diphtheriae* wild-type and ∆*rnj* strains was prepared using RNeasy Mini Kits and RNase-free DNase Sets according to the manufacture’s protocol (Qiagen, Germantown, MD, USA). Briefly, cell pellets harvested from 3 mL log-phase cultures were suspended into 200 μL of chilled 10 mM RNA-free Tris-HCl, pH 8.0. The suspension was added to a fast-protein tube (Q BIOgene, Carlsbad, CA, USA) containing 700 μL RLT buffer (RNeasy Mini Kit, Qiagen), and cells were lysed using a Ribolyser (Hybaid, Cambride, UK). After centrifugation at 13,000× *g*, RNA was purified from the supernatants accordingly. Purified RNA was treated by DNase (Qiagen) and subsequently cleaned by an RNeasy clean up kit (Qiagen). RNA quality was determined by an Agilent 2100 Bioanalyzer (Agilent Technologies) with an Agilent RNA Pico 6000 kit. RNA samples with the RNA integrity number (RIN) values of >8.0 were stored at −80 °C prior to RNA-seq analysis. Three biological replicates of each strain were used for statistical significance.

For RNA-Seq, cDNA libraries were prepared as previously described [37] for each of the biological replicates, and sequencing was performed in the paired-end mode on an Illumina MiSeq. Processing and mapping of the paired-end reads and differential gene expression analysis was performed as previously reported [37]. Genes with an adjusted *p*-value < 0.05 and log_2_ (fold change) above +1.0 or below −1.0, respectively, were considered to be differentially transcribed under the examined conditions. The RNA-seq data have been deposited in the NCBI Gene Expression Omnibus (GEO) database with the accession number of GSE165533.

### 2.7. Reverse Transcription Polymerase Chain Reactions

cDNA was prepared from one microgram of pure RNA according to the manufacture’s instruction (NEB) with random primers and Moloney Murine Leukemia Virus reverse transcriptase (NEB) using a T100 Thermalcycler (BioRad, Hercules, CA, USA). Reverse transcription polymerase chain reactions (RT-PCRs) were performed using a 2.0X Taq RED Master Mix Kit (Genesse Scientific, San Diego, CA, USA) with cDNA as a template and probes for 23S RNA and *rnj* (Table S3).

With cDNA prepared above, real-time qPCR was performed accordingly [39], using iTAQ SYBR green supermix (Bio-Rad) and a T100 Thermalcycler (Bio-Rad). The coding sequence of 23S RNA of *C. diphtheriae* was used as a control. Primers for qRT-PCR are shown in Table S3. Results were analyzed by GraphPad Prism 5.0 (La Jolla, CA, USA).

### 2.8. Detection of Diphtheria Toxin

DT expression and detection were performed according to a published protocol [34]. Briefly, overnight cultures of wild-type and *rnj* mutant strains were used to inoculate fresh cultures with starting OD_600_ of ~0.1. When OD_600_ reached 0.3 at 30 °C, bacterial cultures were treated with 1 mM ethylenediamine-N,N’-bis(2-hydroxyphenylacetic acid) (EDDA; Sigma) for 12 h to induce DT expression. Supernatants were collected for protein precipitation and protein concentration was determined using a BCA Protein assay kit (Thermo Scientific, Waltham, MA, USA). Protein samples were boiled in sample buffer containing 1% SDS prior to SDS-PAGE with 3–20% Tris-glycine gradient gels. DT was detected by immunoblotting with monoclonal antibodies (1:2000, α-DT).

### 2.9. Electron Microscopy

Log-phase cells of *C. diphtheriae* strains were harvested and subject to electron microscopy as previously described [39]. Briefly, cells immobilized on grids were washed five times with distilled water and stained with 1% uranyl acetate for 1 min prior to viewing by a JEOL JEM-1400 electron microscope.

Electron micrographs above were used to measure cell width and length by ImageJ (NIH). For each dimension, 50 individual cells were analyzed. The results were expressed as average with standard deviations as error bars.

### 2.10. Chrome Azurol S (CAS) Assay

Detection of secreted siderophore was performed by a previously published protocol [42] with some modifications. Briefly, corynebacterial cells grown overnight in HI broth at 30 °C were washed in a semi-defined low-iron medium (mPGT) and resuspended in fresh mPGT supplemented with or without 10 μM FeCl_3_ to inoculate new cultures with a starting OD_600_ of 0.05. After 20 h of growing at 30 °C, supernatants obtained by centrifugation were used in a colorimetric assay with chrome azurol S (CAS) to estimate siderophore production according to a published procedure [43]. In brief, obtained supernatants were mixed with CAS solution (3:1 ratio) and incubated at room temperature for 3 h, and absorbance at 630 nm of the resulting solutions were measured using a microplate reader (Tecan M1000). Data from three independent experiments performed in triplicates were expressed as mean with standard deviations. Siderophore production (SP) was estimated by the following equation: SP = (A_r_ − A_s_) × 100/A_r_, where A_r_ is absorbance of reference (CAS solution and un-inoculated broth) and A_s_ is absorbance of sample (CAS solution and culture supernatant).

### 2.11. Caenorhabditis Elegans Killing Assay

*C. elegans* strain N2 was maintained on nematode growth (NG) agar plates containing a bacterial lawn of *E. coli* OP50. Killing assays were performed as previously described [35,44,45], with some modifications. BHI agar plates, supplemented with 25 μg ml^−1^ nalidixic acid and 50 μg ml^−1^ 5-fluoro-2-deoxyuridine (FuDR), were used to grow *C. diphtheriae* from overnight cultures. The plates were incubated at 37 °C for 24 h. Next, L4-stage nematodes were transferred to BHI plates containing *C. diphtheriae* strains and incubated at 25 °C for the remainder of the experiment. Dead nematodes were recorded and removed every 24 h. For each corynebacterial strain, ∼30 nematodes were used, and the assays were repeated at least three times. The Kaplan–Meier method was used for survival analysis, and all statistical analysis was performed with GraphPad Prism 5.0 (La Jolla, CA, USA), with *p*-values <0.05 considered significant. LT_50_, the time required to kill 50% of nematodes, was determined for each bacterial strain.

### 2.12. Statistical Analysis

Statistical analysis was performed using GraphPad Prism 5.0 (La Jolla, CA, USA), with significant difference calculated using the unpaired *t*-test with Welch’s correction. Results were presented as the average of three independent experiments ± standard deviation (SD). A nonparametric, two-tailed value of *p* < 0.05 (*), *p* < 0.01 (**), or *p* < 0.001 (***) was considered significant.

## 3. Results

### 3.1. C. Diphtheriae Encodes Ribonuclease J

**Figure 1:**
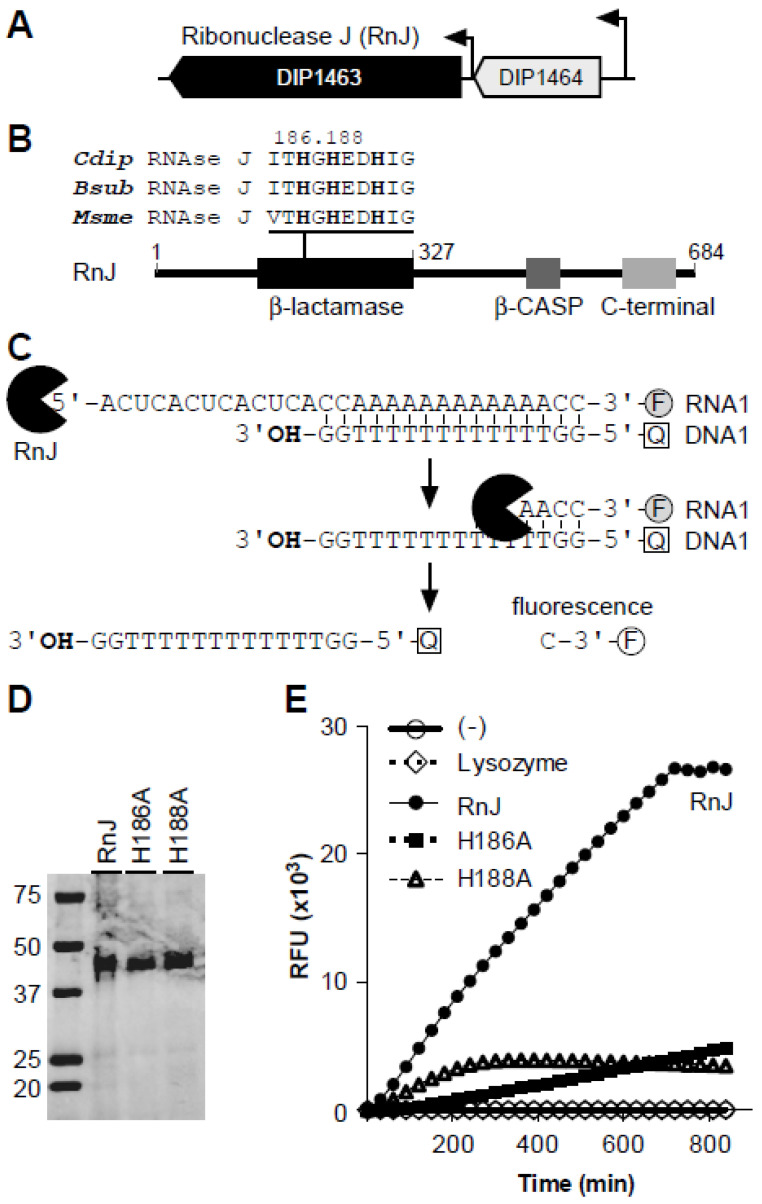
*Corynebacterium diphtheriae* encodes ribonuclease J (RnJ). (**A**) Presented is a diagram of a two-gene locus, in which DIP1463 is predicted to encode ribonuclease J (RnJ). The *rnj* gene is equipped with its own promoter but it is also part of a transcription unit with DIP1464. (**B**) RnJ harbors three domains—β-lactamase, β-CASP, and C-terminal. The β-lactamase region contains catalytic histidine residues in the motif II, which is highly conserved with other ribonuclease J proteins, including *Bacillus subtilis* (Bsub) and *Mycobacterium smegmatis* (Msme); numbers indicate the amino acid positions. (**C**) An RNA/DNA duplex, containing an RNA molecule with a fluorophore (F) at the 3′ (RNA1) and a DNA molecule with a quencher (Q) at the 5′ (DNA1), was used in a real-time fluorescence assay (RT-FeDEx) to probe exoribonuclease activity. Cleavage of RNA1 by DIP1463/RnJ permits fluorescence emission from the released fluorophore; adapted from [41]. (**D**) Purified RnJ recombinant proteins (wild-type and His mutants) were analyzed by SDS-PAGE and stained by Coomassie Blue; molecular mass markers are shown. (**E**) Five hundred nM of the RNA1/DNA1 duplex was mixed with 100 nM of recombinant RnJ proteins. Fluorescence signal was monitored over time. The data are representative of two independent experiments performed in triplicate. Lysozyme and a reaction without RnJ were used as controls.

The predicted ribonuclease J homolog (RnJ) in *C. diphtheriae* NCTC 13,129 genome, DIP1463, is part of a transcriptional unit [37], comprised of DIP1463 and DIP1464 (coding for a 4-hydroxy-tetrahydrodipicolinate synthase and involved in L-lysine biosynthesis) (Figure 1A). Similar to the *Bacillus* RNase J enzymes, the corynebacterial RnJ is predicted to harbor an N-terminal β-lactamase domain, with the conserved catalytic histidine residues His^186^ and His^188^, followed by the β-CASP domain and a C-terminal extension domain (Figure 1B). To examine if corynebacterial RnJ possesses ribonuclease activity, we cloned an RnJ protein, encompassing the β-lactamase domain (residues 1–327) (Figure 1B), in *E. coli* using the expression vector pMCSG7 that appends a hexa-histidine tag to the C-terminus of a cloned protein [40]. Mutant proteins with Ala-substitution mutants of His^186^ and His^188^ were also generated. All recombinant proteins were expressed in and purified from *E. coli* by Ni^2+^ affinity chromatography to homogeneity (see Figure 1D).

Next, we used the purified proteins in a real-time fluorescence detection and assay of exoribonucleases (RT-FeDEx) with an RNA1/DNA1 duplex as a substrate, as previously reported [41]. In this assay, an RNA molecule linked to the fluorophore carboxy-fluorescein (F) at the 3′ end (RNA1) was hybridized to a DNA oligonucleotide containing the quencher carboxytetramethylrhodamine (Q) at the 5′ end (DNA1) (Figure 1C). Incubation of the RNA1/DNA1 duplex with the wild-type RnJ enzyme led to a rapid release of the fluorophore, hence fluorescence over time, whereas no significant increase in the fluorescent signal was visible in the reactions with the two His-to-Ala substitution mutants or lysozyme as control (Figure 1E). Thus, like its counterpart from *Bacillus*, *C. diphtheriae* DIP1463 possesses 5′-3′ exoribonuclease activity that requires the conserved His^186^ and His^188^ catalytic residues; hereafter, we named DIP1463 as ribonuclease RnJ. 

### 3.2. Genetic Disruption of rnj Alters Cell Growth and Morphology

**Figure 2:**
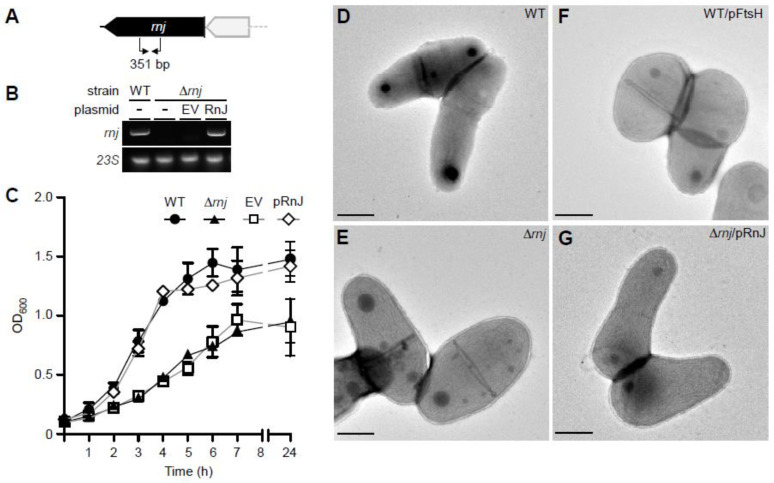
The *C. diphtheriae* ∆*rnj* mutant is defective in cell growth and morphology. (**A**,**B**) An in-frame, non-polar mutant of *rnj* was generated. The presence of *rnj* in the parental, deletion mutant, and complementing strains was determined by RT-PCR using probes (arrows) that detect a 351-bp amplicon; 23S rRNA was used as control. (**C**) Overnight cultures of *C. diphtheriae* indicated strains grown at 30 °C were used to inoculate fresh cultures, and their growth was monitored by OD_600_. (**D**–**G**) Log-phase cells were immobilized on nickel grids, stained with 1% uranyl acetate, and viewed by an electron microscope JEOL1400; scale bars indicate 0.5 μm.

To determine the biological role of RnJ, we constructed an in-frame, nonpolar deletion mutant of *rnj* (∆*rnj*) by a convenient allele-exchange procedure (see Section 2). Using oligonucleotide primers specific for *rnj* (Figure 2A), the generated ∆*rnj* mutant was confirmed by reverse transcriptase (RT)-PCR (Figure 2B). To examine whether *rnj* deletion has any effects on cell growth, the mutant and parental strains were inoculated from overnight cultures, and cell growth over time was monitored by optical density at 600 nm (OD_600_). Compared to the parental strain (WT), the ∆*rnj* mutant displayed a slow growth rate, which was rescued by ectopic expression of *rnj* from a plasmid (Figure 2C). Whereas the doubling time of parental cells was typically 59.03 ± 5.29 min, that of the ∆*rnj* mutant was nearly twice as long (96.65 ± 5.29 min).

We next examined whether the slow growth defect was associated with any alteration in cell morphology using electron microscopy. Corynebacterial cells grown to log-phase were immobilized on nickel grids and stained with 1% uranyl acetate prior to viewing by an electron microscope. Intriguingly, ∆*rnj* mutant cells appeared wider than the parental cells (Figure 2; compare 2D with 2E). This augmentation of cell width was abated when *rnj* was ectopically expressed in the ∆*rnj* mutant (Figure 2G). To quantitatively determine the cell dimension, we employed ImageJ (NIH), measuring cell length and width of 50 cells from electron micrographs taken from parental and mutant cells. As shown in Table 1, the parental cells had an average width and length of 0.64 and 1.26 µm, respectively. The width of the ∆*rnj* mutant increased to 0.99 µm, while its length was similar to that of the parental strain. Prompted by our transcriptomic analysis revealing that corynebacteria devoid of *rnj* contain a higher level of transcripts for the cell division protein FtsH (see below), we examined whether this alteration might contribute to altered cell envelope morphology. Indeed, overexpression of FtsH in the parental strain also caused cell width augmentation, whereas the cell length was comparable to the parental cells (Figure 2F and Table 1). Collectively, these results demonstrate that genetic disruption of *rnj* in *C. diphtheriae* causes cell growth and morphology defects and suggest that the slow growth of the mutant might be linked to an aberrant expansion of the cell envelope width, possibly due to the elevated expression of a morphogen.

### 3.3. Transcriptome Analysis of the rnj Mutant

**Figure 3:**
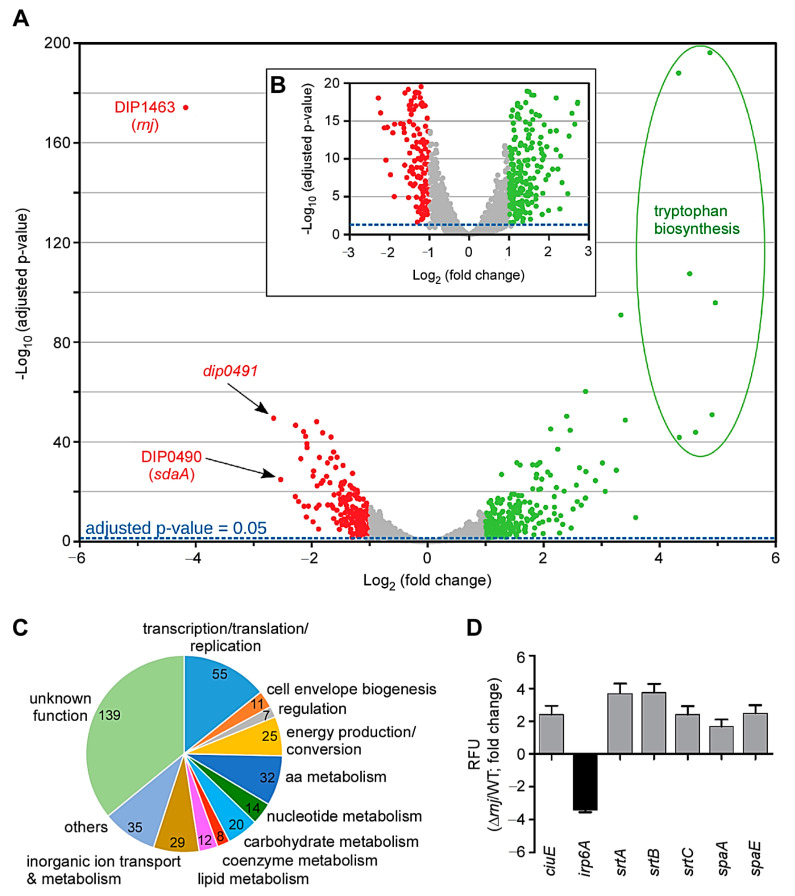
RNA-Seq analysis of the ∆*rnj* mutant. (**A**) Presented is a volcano plot of differentially transcribed genes in the ∆*rnj* mutant. The log_2_ (fold change) (LFC) was plotted against the statistical significance (−log_10_ of the adjusted *p*-value) for each gene. The dashed blue line indicates the negative log_10_ of the adjusted *p*-value of 0.05. All genes above a LFC of +1.0 and below −1.0 are colored in green and red, respectively. Genes predicted to be involved in tryptophan biosynthesis are encircled. Three genes, including the remaining part of the *rnj* transcript in the *rnj* deletion mutant, are labeled in red. (**B**) This insert represents a detail of the volcano plot to better indicate the position of the adjusted *p*-value of 0.05 (dashed blue line). (**C**) Differentially regulated genes with similar categorical functions based on clusters of orthologous groups are shown in a pie chart. (**D**) Some differentially regulated genes in the RNA-seq analysis were confirmed by RT-PCR using RNA samples collected from mid-log phase cells; 23S rRNA was used as control.

In order to decipher how the deletion of RnJ alters cell morphology and decelerates cell growth, we employed RNA sequencing (RNA-seq) to compare global gene expression between the WT and ∆*rnj* mutant strains. Total bacterial RNA was extracted from three independent cultivations of the wild-type and ∆*rnj* mutant strains at mid-logarithmic growth phase and subjected to RNA-seq using Illumina MiSeq. Using a 2-fold cutoff (log_2_-fold change of ±1), 230 genes were found upregulated (Figure 3A; green dots), while only 157 genes were downregulated by *rnj* deletion (Figure 3A; red dots) (Table S1). Among the differentially regulated genes, a large number of genes (139) encoded proteins with unknown functions, while 115 genes coded for proteins related to metabolism (amino acids, nucleotides, carbohydrates, coenzymes, lipids, and inorganic ions), and 55 genes coded for proteins predictably involved in replication, transcription, and translation, (Figure 3C). Interestingly, five pilus-associated genes (coding for sortases SrtA, SrtB, and SrtC, and pilins SpaE and SpaF) were also upregulated (Table S1). The remaining differentially regulated genes encoded transcriptional regulators, phage proteins, transposases, chaperones, and many others. The most upregulated were genes related to tryptophan biosynthesis (8–10-fold increase) (Figure 3A; green circles). In contrast, DIP0491–coding for L-asparaginase– and *sdaA* (L-serine dehydratase encoding gene) were among the most highly downregulated genes. To confirm differential gene expression, we isolated total RNA from the parental and ∆*rnj* mutant strains for qRT-PCR using probes specific for some of the genes listed in Table 2. Figure 3D shows that the expression of *ciuE,* encoding a siderophore-biosynthesis protein [29], was increased by 2-fold, whereas the expression of *irp6A,* encoding a siderophore transporter [29], was decreased nearly 4-fold in the ∆*rnj* mutant. All pilus-associated genes were upregulated in this mutant as expected (Figure 3D).

Intriguingly, *ftsH* was upregulated (log_2_-fold change of +1.26) in the *rnj* mutant. The *ftsH* gene was originally identified in *E. coli* through the isolation of a temperature sensitive cell division mutant, which had elongated cells [46]. In *B. subtilis*, deletion of *ftsH* also resulted in filamentous growth [47]. These findings led us to hypothesize that *ftsH* might be associated with cell division in *C. diphtheriae*. To test this, we engineered a recombinant multi-copy vector expressing *ftsH* under the control of a strong promoter and introduced the resulting plasmid into the wild-type strain. Cell morphology was then analyzed by electron microscopy. As shown in Figure 2F, this strain displayed a phenotype similar to the ∆*rnj* mutant, i.e., augmentation of cell width (see also Table 1), suggesting that the altered cell morphology observed in the ∆*rnj* mutant might be caused by the increased expression of *ftsH* in this mutant.

Taken together, the results support that the RnJ ribonuclease modulates a large number of transcripts in the human pathogen *C. diphtheriae*, which are involved in cell metabolism, fitness, and morphology.

### 3.4. Reduction of Secreted Diphtheria Toxin in the ∆rnJ Mutant

**Figure 4:**
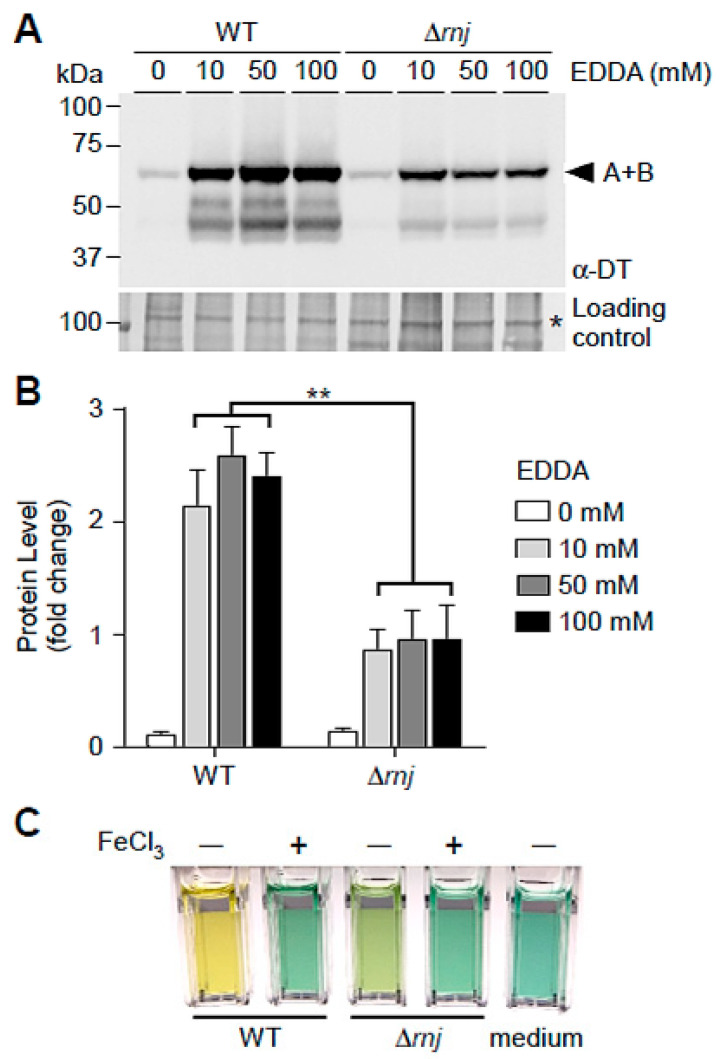
Reduced production of the exotoxin diphtheria toxin in the ∆*rnj* mutant. (**A**) Cells of the *C. diphtheriae* parental strain (WT) or ∆*rnj* mutant strain were treated with various concentrations of the iron chelator ethylene diamine diorthohydroxyphenyl acetic acid (EDDA) to induce expression of diphtheria toxin (DT). Harvested supernatants were subjected to TCA precipitation, and protein samples were immunoblotted with monoclonal antibodies against DT; arrowhead indicates the intact toxin. A loading control band from an immunoblotted membrane stained with Coomassie Blue is marked with an asterisk. The results are representative of four independent experiments. (**B**) Density of DT blots in (**A**) was quantified by ImageJ and analyzed using GraphPad Prism 5.0, with ** indicating *p* < 0.01. (**C**) Bacterial cells were grown in a semi-defined low-iron medium supplemented with 10 μM FeCl_3_. Production of extracellular siderophore was determined by a colorimetric assay using chrome azurol S.

The major virulence factor of *C. diphtheriae* is the exotoxin diphtheria toxin (DT) encoded by a prophage, i.e., the corynephage β [23,35]. DT expression is negatively controlled by the repressor DtxR, whose activation as a repressor requires iron; thus, *tox* expression is induced when iron is limiting, which deactivates DtxR as a repressor [48]. Note that our RNA-seq data revealed no significant changes of *tox* and *dtxR* transcripts in the ∆*rnj* mutant as compared to the parental strain (Table S1). On the contrary, many genes involved in iron transport and metabolism were affected in the ∆*rnj* deletion mutant (Table S1). To investigate this phenomenon further under iron-limiting conditions, cultures of the parental and ∆*rnj* mutant strains were treated with varying amounts of the iron-chelator EDDA (ethylene diamine diorthohydroxyphenyl acetic acid). Cell-free culture supernatants were then obtained for Western blotting analysis with antibodies against DT. In the parental strain without EDDA, the level of DT expression was negligible. However, upon addition of 10, 50, or 100 mM of the chelator, DT level increased dramatically (Figure 4A,B); by comparison, the level of DT expression in the ∆*rnj* mutant was about 2-fold less than that of the parental strain under the same conditions (Figure 4A,B).

**Figure 5:**
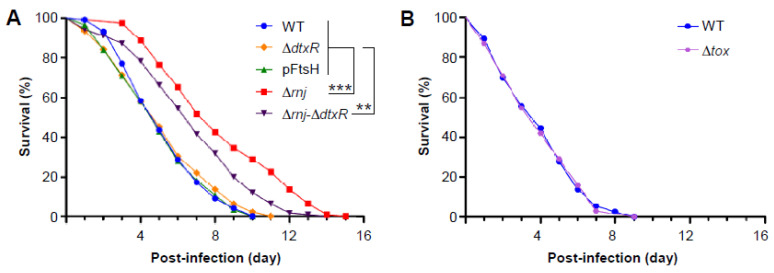
Virulence attenuation of the ∆*rnj* mutant in a *C. elegans* model of infection. (**A**) L4-stage nematodes of strain N2 were fed on corynebacteria of strains NCTC 13129 (WT, blue circles), NCTC 13129 overexpressing FtsH (green triangles), ∆*rnj* mutant (red squares), ∆*dtxR* mutant (orange diamonds), or ∆*rnj*-∆*dtxR* mutant (black triangles). Dead nematodes were recorded and removed every 24 h. (**B**) A similar experiment as described in (**A**) was carried out with the WT and ∆*tox* mutant (purple hexagons) strains. The results are presented as survival percentage, and statistical analysis was performed using GraphPad Prism 5.0. Asterisks ** and *** indicate *p* values of <0.01 and <0.001, respectively.

Previous studies show that under iron-deprivation *C. diphtheriae* secretes corynebactin, a siderophore that scavenges extracellular iron, which correlates with production of DT [49,50]. To examine if siderophore production is reduced under iron-depleted conditions, we grew the ∆*rnj* mutant and wild-type cells in a semi-defined low-iron medium, called mPGT [51], supplemented with or without 10 μM FeCl_3_. Production of extracellular siderophore from the culture medium was determined by the chrome azurol S (CAS) assay, as previously reported [42,43]. As shown in Figure 5C, production of extracellular siderophore in the ∆*rnj* mutant was significantly reduced as compared to the wild-type strain (see also Table 3). Taken together, the results demonstrate that deletion of *rnj* causes reduced production of secreted diphtheria toxin and siderophore.

### 3.5. Virulence Attenuation of the ∆rnj Mutant in a Caenorhabditis Elegans Model of Infection

Finally, we went on to address the most critical question of whether the reduced DT production, altered cellular fitness, morphology, and metabolism observed in the ∆*rnj* mutant affect bacterial virulence using a previously established model host of *C. elegans* for *C. diphtheriae* infection [35,44]. In this model, batches of L4-stage nematodes of strain N2 were fed with corynebacteria, and the survival of nematodes was recorded every 24 h over time. As shown in Figure 5A (red squares), the ∆*rnj* mutant exhibited a significant delay in the killing of nematodes, with LT_50_ (time required to kill 50% of the animals) of seven days, as compared to the wild-type strain (blue circles), LT_50_ of which was less than five days. As expected, a mutant devoid of the repressor DtxR displayed a similar virulence phenotype as the wild-type strain (Figure 5A; ∆*dtxR*, orange diamonds). However, a mutant lacking both *rnj* and *dtxR* was markedly attenuated as compared to the wild-type strain, but this attenuation was less than that of the ∆*rnj* mutant itself (Figure 5A; ∆*rnj*-∆*dtxR*, black triangles). To examine whether cell width augmentation caused by increased expression of FtsH affects bacterial virulence, we used a strain overexpressing *ftsH* (see Figure 2) in this *C. elegans* survival assay. Importantly, the altered cell morphology did not make any difference in the rate of nematode killing as compared to the parental strain (Figure 5A). Lastly, to determine how DT affects nematode killing, we exposed *C. elegans* to the ∆*tox* mutant of *C. diphtheriae*. Strikingly, the rate of survival of nematodes exposed to the ∆*tox* mutant of *C. diphtheriae* was the same as the wild-type corynebacteria (Figure 5B). These results demonstrate that the genetic disruption of *rnj* attenuates corynebacterial virulence by affecting some mechanism distinct from diphtheria toxin-mediated killing.

## 4. Discussion

RNA turnover works in conjunction with transcriptional mechanisms in bacteria to permit rapid adaptation of protein synthesis to environmental challenges. While a wide variety of RNases, which partake in mRNA maturation and degradation and in turn regulation of gene expression, have been identified and characterized in many bacterial systems [5], to date there is a scarcity of information regarding these processes in the actinobacterium *C. diphtheriae*—a human pathogen of major significance that was contained in the past century by aggressive vaccination around the world. Bioinformatics and the biochemical and genetic studies presented here demonstrate that the *C. diphtheriae* DIP1463 encodes a member of the class D ribonuclease, RNase J, and that this ribonuclease acts to modulate the expression of a large number of genes involved in many cellular processes, including cell division, metabolism, and pathogenicity.

Our biochemical work using a recombinant RnJ protein demonstrated that the exoribonuclease activity of RnJ is confined within the N-terminal 327-amino acid region that comprises a widely conserved β-lactamase domain (Figure 1A). Previously, four conserved motifs—namely, I–IV—have been identified in the β-lactamase domain of RNase J proteins, and the motif II is known to harbor several histidine residues that appear to be critical for catalytic activity [52]. Consistent with this picture, our mutational analysis demonstrated that alanine substitution of the His^186^ and His^188^ residues within motif II abrogates the exoribonuclease activity of *C. diphtheriae* RnJ (Figure 1D). The results also imply that the β-CASP and C-terminal domains are not required for RnJ exoribonuclease activity.

The comparative transcriptome analysis by RNA-seq reported here revealed that ribonuclease RnJ is a central player of mRNA turnover in *C. diphtheriae*. The deletion of *rnj* induces differential expression of a sizable number of transcripts (387 when a 2-fold cutoff, i.e., log_2_-fold change of ±1, is implemented), among which L-tryptophan biosynthesis genes are highly expressed in the ∆*rnj* mutant (Figure 3). It is noteworthy that the RNase J1-dependent degradation of *trp* leader RNA has been reported in *B. subtilis* [53], although it is not clear if similar mechanisms would be observed in *C. diphtheriae*. Intriguingly, the transcript level of *ftsH*, coding for an inner membrane AAA^+^-type protease, is also elevated in the ∆*rnj* mutant, which exhibits a significantly wider cell width compared to the parental strain (Figure 2). Given the pivotal role of FtsH protease in cellular quality control and several critical regulatory circuits [54], we surmised that the augmentation of cell width in the ∆*rnj* mutant might be due to aberrant expression of *ftsH*. Indeed, *ftsH* overexpression in the parental strain leads to a similar alteration in cell morphology (Figure 2F).

Importantly, while it is striking that the ∆*rnj* mutant is attenuated in the *C. elegans* model of infection (Figure 5), this attenuation is not attributable to the aberrant cell morphology: an engineered strain with elevated FtsH mimics the altered cell morphology phenotype, but it remains as virulent as the wild-type strain (Figure 5). Even more striking is our demonstration here that although the production of diphtheria toxin is visibly reduced in the absence of *rnj*, and DT is one of the most significant virulence factors for human disease, the toxin itself does not play a role in nematode killing by *C. diphtheriae* (Figure 5B). This rather unexpected finding poses important puzzles about the virulence factors encoded by corynebacteria, whether toxigenic or not, and the role that RNase J plays in regulating some of them. A tentative clue about the candidate factors comes from a small list of metabolic genes affected by RNase J depletion—namely, the upregulation of trp operon genes required for tryptophan biosynthesis and downregulation of *sdaA* coding for L-serine dehydratase that converts serine to pyruvate (Figure 3 and Table S1). Importantly, tryptophan biosynthesis utilizes several key intermediate metabolites: glutamine, serine, and phosphoribosyl pyrophosphate, the essential precursor to nucleotide biosynthesis [55]. An uncontrolled capacity for tryptophan biosynthesis in the ∆*rnj* mutant may have a consequence of depleting cells of these essential nutrients while also producing several byproducts, e.g., glyceraldehyde-3-phosphate, at an elevated rate. It is tempting to speculate that this metabolic exhaustion and imbalance could hamper corynebacterial growth within the nematode. As the number of genes affected by RnJ ribonuclease are far too many, unveiling the critical pathogenic factors will require a systematic approach to probe the various cellular pathways that are found abnormal in the ∆*rnj* mutant. Fortunately, this should not be too forbidding considering the convenience of the nematode model system and the facile genetic systems available to perturb the candidate factors in *C. diphtheriae* that are involved in the aforementioned critical metabolic pathways, as well as iron acquisition, cell surface structure, and assembly and gene regulation.

Finally, while it is clear that RnJ is a major ribonuclease in *C. diphtheria* mRNA metabolism, our bioinformatics analysis of the *C. diphtheriae* strain NCTC 13129 genome reveals that there is just one copy of RNase J in this organism and that the bacterium also encodes an RNase E homolog. Considering the established critical role of RNase E in many bacteria, and the overlapping function of RNase J in mRNA metabolism, it will be revealing to compare and contrast the regulons controlled by RnJ and RNase E in *C. diphtheriae*. Together, this will illuminate the conserved and unique aspects of post-transcriptional control of genes of bacterial pathogens involved in cell division, nutrient uptake, and virulence.

## Figures and Tables

**Table 1:** Cell dimension of corynebacterial strains.

Strains	Width (µm)	Length (µm)
WT	0.64 ± 0.06	1.26 ± 0.40
∆*rnj*	0.99 ± 0.13	1.39 ± 0.36
WT/pFtsH	0.90 ± 0.07	1.32 ± 0.33

**Table 2:** Differential expression of representative genes involved in iron or heme transport in the ∆*rnj* mutant determined by RNA-seq, using a 2-fold cutoff (log2-fold change of ±1).

Gene Name	Function	Log_2_-Fold Change
*iutD*	Putative ABC-type iron protein	1.03
*ciuC*	Iron transport system membrane protein	1.18
*ciuE*	Corynebactin biosynthetic gene	1.24
*sufB*	Fe–S cluster assembly protein	1.33
*sufR*	Iron–sulfur cluster biosynthesis transcriptional regulator	1.56
*piuB*	Iron-uptake factor	1.83
*irp4*	DtxR-dependent, iron-regulated promoter/operator	2.65
*irp6A*	Ferrisiderophore receptor (putative ABC transporter)	−1.30
*irp5*	DtxR-dependent, iron-regulated promoter/operator	−1.33

**Table 3:** Siderophore production of *C. diphtheriae* strains in high- or low-iron media.

Strains	+Fe^3+^		−Fe^3+^	
	OD_600_	Siderophore Production (%)	OD_600_	Siderophore Production (%)
WT	3.38 ± 0.08	19.30 ± 2.99	2.62 ± 0.29	67.77 ± 2.90
∆*rnj*	2.92 ± 0.31	20.42 ± 1.97	2.38 ± 0.23	44.49 ± 3.43

## Data Availability

The RNA-seq data have been deposited in the NCBI Gene Expression Omnibus (GEO) database with the accession number of GSE165533.

## Supplementary Materials

The following are available online at https://www.mdpi.com/2076-2607/9/2/389/s1, Table S1: Differential gene expression analyzed by RNA-seq in the *rnj* mutant; Table S2: Bacterial strains and plasmids used in this study; Table S3: Primers used in this study.
