# Supplementary material for: Ribonuclease J-Mediated mRNA Turnover Modulates Cell Shape, Metabolism and Virulence in Corynebacterium diphtheriae
Source: Microorganisms. 2021 Feb 14;9(2):389. doi: 10.3390/microorganisms9020389 (PMC7917786; doi:10.3390/microorganisms9020389)
Supplement: Supplementary file 1 [file microorganisms-09-00389-s001.zip › Supplementary Information.docx]

**Ribonuclease J mediated mRNA turnover modulates cell shape, metabolism and virulence in *Corynebacterium diphtheriae***

**Truc Thanh Luong,^1,2*^ Minh Tan Nguyen,^1,3*^ Yi-Wei Chen,^1*^ Chungyu Chang,^1^ Ju Huck Lee,^2,4^ Manuel Wittchen,^5^ HyLam Ton-That,^6^ Melissa Cruz,^2^ Danielle A. Garsin,^2^ Asis Das,^7†^ Andreas Tauch,^5^ and Hung Ton-That^1,8†^**

^1^*Division of Oral Biology & Medicine, School of Dentistry, University of California Los Angeles, CA, USA*; ^2^*Department of Microbiology & Molecular Genetics, University of Texas Health Science Center, Houston, TX, USA*; *^3^NTT Hi-Tech Institute, Nguyen Tat Thanh University, Ho Chi Minh City, Vietnam;* ^4^*Biological Resource Center, Korea Research Institute of Bioscience and Biotechnology, Jeollabuk-do, Republic of Korea*; *^5^Center for Biotechnology (CeBiTec), Bielefeld University, Bielefeld, Germany*; ^6^*Department of Chemistry, University of California at Irvine, Irvine, CA, USA;* ^7^*Department of Medicine, Neag Comprehensive Cancer Center, University of Connecticut Health Center, Farmington, CT, USA;* ^8^*Molecular Biology Institute, University of California Los Angeles, CA USA*

*Equal contribution

Running title: Pleotropic effects of corynebacterial RNase J mutation

†To whom correspondence should be addressed:

Hung Ton-That, [htonthat@dentistry.ucla.edu](mailto:htonthat@dentistry.ucla.edu); Asis Das, [adas@ucla.edu](mailto:adas@ucla.edu)

**Supplemental Table S1:** Differential gene expression analyzed by RNA-seq in the *rnj* mutant (attached)

**Supplemental Table S2:** Bacterial strains and plasmids used in this study

| **Strain and Plasmid** | **Description** | **Reference** |
| --- | --- | --- |
| *Strain* |  |  |
| *C. diphtheriae* NCTC 13129 | Type strain | [1] |
| *C. diphtheriae* ∆*rnj* | Derivative of NCTC13129; deletion of DIP1463 | This study |
| *C. diphtheriae* ∆*dtxR* | Derivative of NCTC13129; deletion of *dtxR* | [2] |
| *C. diphtheriae* ∆*rnj*-∆*dtxR* | Derivative of NCTC13129; deletion of *rnj* & *dtxR* | This study |
| *C. diphtheriae* ∆*tox* | Derivative of NCTC13129; deletion of *tox* | [3] |
| *Plasmid* |  |  |
| pCGL0243 | *E. coli*/*Corynebacterium* shuttle vector; Kan^R^ | [4] |
| pRnJ | Derivative of pCGL0243, expressing *C. diphtheriae* RnJ under its native promoter | This study |
| pFtsH | Derivative of pCGL0243, expressing *C. diphtheriae* FtsH under the control of the *C. diphtheriae spaA* promoter | This study |
| pK19*mobsacB* | Conjugative plasmid for deletion mutant | [5] |
| p∆*rnj* | Derivative of pK19*mobsacB*, carrying a *rnj* deletion cassette | This study |
| p∆*dtxR* | Derivative of pK19*mobsacB*, carrying a *dtxR* deletion cassette | [2] |
| pMCSG7 | Expression vector | [6] |
| pHis6-RnJ | pMCSG7 expressing the β-lactamase domain of RnJ (residues 1-327) | This study |
| pHis6-H186A | Derivative of pHis6-RnJ harboring H186A | This study |
| pHis6-H188A | Derivative of pHis6-RnJ harboring H188A | This study |

**Supplemental Table S3:** Primers used in this study

| **Primer** | **Sequence** | **Used for** |
| --- | --- | --- |
| EcoRI-DIP1464-up-F | CCGGAATTCGAAAATGTCGATATTTCG | ∆*rnj* |
| DIP1464-DIP1462-R | CCCATCCACTAAACTTAAACAGCTACGAGGTTCAGTCAT | ∆*rnj* |
| DIP1464-DIP1462-F | TGTTTAAGTTTAGTGGATGGGCGAAAATGATCCATATCG | ∆*rnj* |
| BamHI-DIP1462-dn-R | CGCGGATCCGTCATGGTCTCCAGCTTAG | ∆*rnj* |
| pRnJ-SalI-F | ACGCGTCGACATGGTTGTCGGTAGGTGC | pRnJ |
| pRnJ-BamHI-R | CCCGGATCCGGG CTACTGACTTTCGCGGCTC | pRnJ |
| pFtsH-HindIII-A | CCCAAGCTTGGGGCAATATTTG GGGTGCACAG | pFtsH |
| pFtsH-B | CTTTTTGTTGTTcatACAATCCCTCAACT | pFtsH |
| pFtsH-C | AGTT GAGGGATTGTatgAACAACA AAAAGACCC | pFtsH |
| pFtsH-XhoI-D | CGCGAGCTCGCGttaCTCGTCAGTTTCTTCGACTC | pFtsH |
| LIC-RnJ_981_-F | TACTTCCAATCCAATGCAatgACTGAAC CTCGTAGCC | pHis6-RnJ |
| LIC-RnJ_981_-R | TTATCCACTTCCAATGTTAGATCAAAGTTGGAGCGACGTCTG | pHis6-RnJ |
| LIC-H186A-F | TGATTACTGCCGGTCACGAAGAC | pHis6-H186A |
| LIC-H186A-R | CCAAAGCATCAACTTTGTCG | pHis6-H186A |
| LIC-H188A-F | CACGGTGCCGAAGACCACATC | pHis6-H188A |
| LIC-H188A-R | AGTAATCACCAAAGCATCAAC | pHis6-H188A |
| RT-23sRNA-F | GCCGCTTTAATGGGCGAAC | RT-PCR |
| RT-23sRNA-R | GGGACTAGTGATCCGGCACC | RT-PCR |
| RT-rnj-F | ACCACTCGATTCCTGATTGC | RT-PCR |
| RT-rnj-R | CTACTGACTTTCGCGGCTCGC | RT-PCR |
| RT-ciuE-F | CGTCGTCGTAGGAGGACACC | RT-PCR |
| RT-ciuE-R | CATTGGAATGGGTGCACGGG | RT-PCR |
| RT-irp6A-F | AAGGCAGCCGAGCAAAATGG | RT-PCR |
| RT-irp6A-R | CTGCTCGCCAAGAACAGCAC | RT-PCR |
| RT-irp6B-F | CGATTGTTTTGGGCTGGGTG | RT-PCR |
| RT-irp6B-R | AGCGCCTGGAACAAAAACAC | RT-PCR |
| RT-srtA-F | CGTCGGTAGTAGGCTTCCCG | RT-PCR |
| RT-srtA-R | GGTGGGCGTCGATAGTAGGG | RT-PCR |
| RT-srtB-F | AGATCAAGAAGGGCGACCCG | RT-PCR |
| RT-srtB-R | TGATGCCATAGGGGGTGCAG | RT-PCR |
| RT-srtC-F | CTCGTGATCCTTGGGGTGCT | RT-PCR |
| RT-srtC-R | TCAAAGATGGGGCCGGTTGT | RT-PCR |
| RT-spaA-F | CACACCAGCCCTCTACCTCG | RT-PCR |
| RT-spaA-R | GCTCAGACAAAGCCTGGTGC | RT-PCR |
| RT-spaE-F | TTCCTGGTGGCGGTTCCTTT | RT-PCR |
| RT-spaE-R | GCGTTCTTTCTCATCGCCCG | RT-PCR |

^(a)^ Underlined are the restriction sites in the primers.

**References**

1. Cerdeno-Tarraga, A.M.; Efstratiou, A.; Dover, L.G.; Holden, M.T.; Pallen, M.; Bentley, S.D.; Besra, G.S.; Churcher, C.; James, K.D.; De Zoysa, A., et al. The complete genome sequence and analysis of *Corynebacterium diphtheriae* NCTC13129. *Nucleic Acids Res* **2003**, *31*, 6516-6523.

2. Wittchen, M.; Busche, T.; Gaspar, A.H.; Lee, J.H.; Ton-That, H.; Kalinowski, J.; Tauch, A. Transcriptome sequencing of the human pathogen *Corynebacterium diphtheriae* NCTC 13129 provides detailed insights into its transcriptional landscape and into DtxR-mediated transcriptional regulation. *BMC Genomics* **2018**, *19*, 82, doi:10.1186/s12864-018-4481-8.

3. Reardon-Robinson, M.E.; Osipiuk, J.; Jooya, N.; Chang, C.; Joachimiak, A.; Das, A.; Ton-That, H. A thiol-disulfide oxidoreductase of the Gram-positive pathogen *Corynebacterium diphtheriae* is essential for viability, pilus assembly, toxin production and virulence. *Mol Microbiol* **2015**, *98*, 1037-1050, doi:10.1111/mmi.13172.

4. Ton-That, H.; Schneewind, O. Assembly of pili on the surface of *Corynebacterium diphtheriae*. *Mol Microbiol* **2003**, *50*, 1429-1438, doi:10.1046/j.1365-2958.2003.03782.x.

5. Schafer, A.; Tauch, A.; Jager, W.; Kalinowski, J.; Thierbach, G.; Puhler, A. Small mobilizable multi-purpose cloning vectors derived from the *Escherichia coli* plasmids pK18 and pK19: selection of defined deletions in the chromosome of Corynebacterium glutamicum. *Gene* **1994**, *145*, 69-73.

6. Stols, L.; Gu, M.; Dieckman, L.; Raffen, R.; Collart, F.R.; Donnelly, M.I. A new vector for high-throughput, ligation-independent cloning encoding a tobacco etch virus protease cleavage site. *Protein Expr Purif* **2002**, *25*, 8-15.
